# Identification of *Anaplasma marginale*, *Babesia bovis* and *Babesia bigemina* resistance alleles in Crioulo Lageano cattle using PCR-SBT and BoLA-DRB3 gene sequencing

**DOI:** 10.3389/fvets.2023.1256928

**Published:** 2023-09-15

**Authors:** Mariana da Silva Casa, Guillermo Giovambattista, Graziela Vieira Fonteque, Ellen Lara Miguel, Carla Ivane Ganz Vogel, Luiz Claudio Miletti, Shin-Nosuke Takeshima, Joandes Henrique Fonteque

**Affiliations:** ^1^Graduate Program in Animal Science (PPGCA), State University of Santa Catarina (UDESC), Lages, Brazil; ^2^Facultad de Ciencias Veterinarias UNLP, IGEVET–Institute of Veterinary Genetics, La Plata, Argentina; ^3^Scientific Initiation Program, State University of Santa Catarina (UDESC), Lages, Brazil; ^4^Department of Animal Production and Food, State University of Santa Catarina (UDESC), Lages, Brazil; ^5^Department of Food and Nutrition, Faculty of Human Life, Jumonji University, Niiza, Saitama, Japan; ^6^Department of Veterinary Medicine, State University of Santa Catarina (UDESC), Lages, Brazil

**Keywords:** allele, Creole breed, PCR, *Anaplasma*, *Babesia*

## Abstract

**Introduction:**

The BoLA-DRB3 gene in cattle is associated with tolerance to several infectious diseases, such as neosporosis, dermatophilosis, leukosis, and mastitis.

**Methods:**

This study used PCR-SBT and BoLA-DRB3 gene sequencing to determine the association between the presence or absence of *Anaplasma marginale*, *Babesia bovis*, and *Babesia bigemina* infections in 208 Crioulo Lageano cattle and alleles present in the population. The chi-square test and odds ratio analysis were employed to establish the association.

**Results:**

Of the BoLA-DRB3 gene alleles present in the population, two alleles were significantly associated with resistance to *A. marginale* infections: *BoLA-DRB3001:01* (*p* < 0.001; OR = 0.224), which had a frequency of 7.93%, and *BoLA-DRB3024:06* (*p* = 0.007; OR < 0.00001), which had a frequency of 0.72%. Regarding *B. bovis* infection, the BoLA-DRB3*011:01 allele (*p* = 0.002; OR = 0.271) had a frequency of 6% in the population and was associated with resistance to the infection. None of the alleles was associated with resistance to infection by *B. bigemina*.

**Discussion:**

The Crioulo Lageano breed has alleles that may confer resistance against infection by *A. marginale* and *B. bovis*.

## 1. Introduction

Locally adapted cattle breeds are crucial for sustainability and food security. Their genetic diversity is especially important in light of environmental challenges like climate change, as it can help improve production characteristics and meet consumer demands (1). Additionally, thanks to such genetic variability, these breeds offer greater resistance to various infectious diseases (2).

Anaplasmosis, caused by *Anaplasma marginale*, is among the most prevalent diseases in cattle and causes enormous economic losses due to the high morbidity and mortality and costs of its control (3). Similarly, babesiosis, caused by *Babesia* sp., leads to high morbidity and mortality when clinically manifested, as well as loss of productive efficiency, generating great economic losses (4). It is estimated that in Brazil these two diseases caused by *Rhipicephalus microplus* (Canestrini, 1888) ticks causes losses of around 3.24 million dollars (5).

The high costs of treatments and the loss of animals affected by anaplasmosis and babesiosis require new alternatives to control these diseases, and one of the most promising alternatives is the selection of animals with genotypes of resistance to clinical diseases (6, 7). These animals are important sources of genetic material for the improvement of herds susceptible to hemoparasites. In this regard, several studies have proven the importance of the BoLA-DRB3 gene with resistance to various infectious diseases (8–10).

The major histocompatibility complex (MHC) is characterized by an intimate relationship between genes that play a role in resistance/susceptibility to infectious diseases, in addition to acting on the innate and adaptive immune response (11). In cattle, MHC (BoLA – bovine leukocyte antigen) is located on chromosome 23 (12) and its genes are grouped into three classes, I, II, and III, according to their function (13). Class II genes are the most studied and are divided into two separate regions of the chromosome. These two parts are known as IIa and IIb (12, 14).

Class IIa has two sub-regions, one composed of the DQ family genes (DQA and DQB) and the other of the DR family genes (DRA and DRB) (15). At least three genes of the DRB family are described in the bovine species: DRB1, which is considered a pseudogene; DRB2, which is little expressed; and DRB3, which is described as highly expressed and polymorphic (16) and associated with tolerance to several diseases, such as neosporosis (17), dermatophilosis (18), leukosis (19, 20), and mastitis (21).

The Crioulo Lageano breed has already accumulated almost five centuries of natural selection and presents characteristics related to resistance to diseases and parasites (22). The challenge of extreme climate conditions during the rigorous winters of the South region of Brazil, the lack of food, predators, and abandonment in vast rural areas are believed to have contributed to the formation of a breed adapted to the environmental conditions of regions of altitude and the subtropical climate (23).

Thus, the presence of resistance alleles to babesiosis and anaplasmosis in locally adapted breeds, such as the Crioulo Lageano breed, associated with the productive potential, demonstrates their importance as a source of genetic material for other herds. Thus, if the resistance of animals to hemoparasites can be increased, reducing treatment costs, loss of animals, and the presence of drug residues in products of animal origin, ensuring sustainable production from locally adapted breeds. For this reason, the aim of this work was to determine resistance BoLA-DRB3 alelles for anaplasmosis and babesiosis infections.

## 2. Materials and methods

### 2.1. Animals and sample acquisition

A total of 208 DNA samples from Crioulo Lageano cattle, including young and adult animals, males and females, from all available categories (bulls, cows, calves, and heifers), from *in situ* breeding properties, all located in the mountainous region of Santa Catarina, South Brazil, with animals exposed to the same climatic and pasture conditions were used. This breed originated from *Bos taurus* lineages have high meat quality and is very rustic with great adaptability to hostily conditions (24).

Blood samples from which DNA was extracted were collected for a previous study approved by CETEA Protocol No. 2461171115.

### 2.2. Physical examination

A physical examination was performed to verify clinical signs compatible with clinical diseases. Heart rate (HR), respiratory rate (RR), ruminal movements (RM), rectal temperature, and mucosal color were measured.

### 2.3. Molecular analysis

First a single multiplex-PCR techniques were used to amplify DNA from *A. marginale*, *B. bovis*, and *B. bigemina*. This reaction included for *A. marginale*, a set of primers based on the MSP5 gene (MSP5 F: 5’-CGC AGA TCT AGC AAA ATC GGC GAG AGG TTT ACC ACT TC-3’e MSP5 R: 5’-GCG CTG CAG TGG CGC AAA ATG CCC GAC ATA CC -3′), whereas for *B. bovis* and *B. bigemina* were those described by Figueroa et al. (25) (BoF: 5 -CAC GAG GAA GGA ACT ACC GAT GTT GA-3 and BoR: 5 - CCA AGG AGC TTC AAC GTA CGA GGT CA-3, for *B. bovis* and BiIA: 5’-CAT CTA ATT TCT CTC CAT ACC CCT CC-3′ e BiIB: 5’-CCT CGG CTT CAA CTC TGA TGC CAA AG −3′). Then a second nested-PCR (n-PCR) was performed for *B. bovis* and *B. bigemina* according to Figueroa et al. (25) BoFN 5 - TCA ACA AGG TAC TCT ATA TGG CTA CC-3 e BoRN 5 -CTA CCG AGC AGA ACC TTC TTC ACC AT-3 for *B. bovis* and (BiIAN: 5’-CGC AAG CCC AGC ACG CCC CGG TGC-3′ e BiIBN: 5’-CCG ACC TGG ATA GGC TGT GTG ATG-3′). The Multiplex-PCR reaction was performed in a final volume of 25 μL of solution with 1 U of Taq Polymerase enzyme GoTaq™ Hot Start Polymerase (Promega, WI, United States), 8.5 pmoles of each primer for each agent, 0.2 mM nucleotides (dNTPs), 3.5 mM magnesium chloride, 5 μL 5X Green GoTaq™ Flexi Buffer (Promega, WI, USA), and 3 μL of DNA (concentration between 20 and 100 ng/μL). Positive controls, using DNA samples donated by Empresa Brasileira de Pesquisa Agropecuária (Embrapa) from experimentally infected animals for each agent and negative controls (that uses water instead of DNA), were used for each reaction.

The temperature conditions applied in the thermocycler (BIOCYCLER™, USA) for the two reactions involved initial denaturation at 95°C for 2 min, followed by 30 cycles of 94°C for 1 min, 54.2°C for 1 min, and 73°C for 1 min, and also a final extension at 73°C for 7 min.

The electrophoresis of amplification products was carried out in a horizontal tank, on a 2% agarose gel added with Unisafe Dye 20,000x (Uniscience, United States). A 100-bp molecular weight marker was used as a standard to determine the size of the sample bands. The electrical source conditions were 140 V and 400 mA for 1 h, with visualization by exposure to ultraviolet light. Bands with a size close to 458 bp for *A. marginale*, 350 bp for *B. bovis*, and 278 bp for *B. bigemina* were considered positive in the first reaction, and bands with a size of approximately 290 bp for *B. bovis* and 170 bp for *B. bigemina* were considered positive in the second reaction (Table 1).

**Table 1:** Sizes of bands in agarose gel 2% for each agent in first and second PCR reactions.

Size of bands on 2% agarose gel	Agent		
	*Anaplasma marginale*	*Bebisia bovis*	*Bebisia bigemina*
First Mulptiplex-PCR reaction	458pb	350pb	278pb
Nested-PCR reaction	-	290pb	170pb

### 2.4. BoLA-DRB-3.2 gene genotyping

Amplification of the BoLA-DRB3 gene second exon was performed through single-step PCR, with primers HLO30 (5′-ATC CTC TCT CTG CAG CAC ATT TCC-3′) and HLO32 (5′-TCG CCG CTG CAC AGT GAA ACT CTC-3′) (26, 27).

The reaction conditions consisted of initial denaturation at 95°C for 5 min, followed by 35 cycles of 95°C for 1 min, 65°C for 1 min, and 72°C for 1 min, and a final extension at 72°C for 10 min. The electrophoresis of amplification products was carried out on a 1.5% agarose gel added with Unisafe Dye 20,000x (Uniscience, United States). A 100 bp molecular weight marker was used as a standard to determine the size of the sample bands. The electrical source conditions were 100 V and 400 mA for 40 min and visualization by exposure to ultraviolet light. The obtained fragment had a size of 284 bp from exon 2 of the BoLA-DRB3 gene. The products of this PCR reaction were then sent to a specialized company (ACTGene – Rio Grande do Sul, Brasil) for sequencing by Sanger method, for the exact identification of alleles. Raw sequence data were analyzed using Assign 400ATF ver. 1.0.2.41 software (Conexio Genomics, Fremantle, Australia) as described by Takeshima et al. (28), and using a database of all BoLA-DRB3 alleles described so far in the IPD-MHC.^1^ Using this information, the software identify the genotype that explain the multiple polymorphic sites within the BoLA-DRB3 second exon.

### 2.5. Statistical analysis

Allele frequency and number of alleles for BoLA-DRB3 gene was obtained by direct counting. For association analysis, animals were grouped in positive and negative for each pathogen, considering that all animals were equaly exposed. Then, the association between each allele with the infection by each pathogen was determined using the chi-square test (*p* < 0.05), followed by the odds ratio analysis, with SigmaPlot12 software (SigmaPlot version 12.0 for Windows, Systat Software Inc., San Jose, CA, United States). Alleles with OR < 1 are considered resistance alleles and those with OR > 1 are considered susceptibility alleles.

## 3. Results

The clinical examination of the samples showed that no Crioulo Lageano animal had clinical signs compatible with anaplasmosis or babesiosis. Table 2 shows the proportions of occurrence of infections for the 208 Crioulo Lageano animals based on the molecular diagnosis performed for the agents *A. marginale*, *B. bovis*, and *B. bigemina*.

**Table 2:** Absolute and relative proportion of Crioulo Lageano animals positive and negative for the agents *Anaplasma marginale*, *Babesia bovis*, and *Babesia bigemina*.

	*Anaplasma marginale*		*Babesia bovis*		*Babesia bigemina*	
Proportion	Positive	Negative	Positive	Negative	Positive	Negative
Absolute	165	43	144	64	127	81
Relative	79.3%	20.7%	69.2%	30.8%	61.0%	39.0%

The association analysis results based on the chi-square test and the odds ratio were summarized in Table 3. These analysis showed that among the alleles of the BoLA-DRB3 gene found in the population, BoLA-DRB3*001:01 (*p* < 0.001; OR = 0.224), with a frequency of 7.93% in the population, and BoLA-DRB3*024:06 (*p* = 0.007; OR < 0.00001), with a frequency of 0.72%, are significantly associated with resistance to *A. marginale* infection in the Crioulo Lageano breed.

**Table 3:** Chi-square (*Χ*^2^) and Odds ratio analysis between BoLA-DRB3 alleles and presence/absence of the agents *Anaplasma marginale*, *Babesia bovis*, and *Babesia bigemina*, to determine resistance/susceptibility alleles for each agent in the Crioulo Lageano cattle.

BoLA-DRB3 allele	*Anaplasma marginale*		*Babesia bovis*		*Babesia bigemina*	
	*Χ*^2^ (value of *p*)	Odds ratio	*Χ*^2^ (value of *p*)	Odds ratio	*Χ*^2^ (value of *p*)	Odds ratio
DRB3*1801	0.152 (0.697)	1.269	3.739 (0.053)	0.513	0.017 (0.895)	0.991
DRB3*2801	0.477 (0.490)	0.705	0.088 (0.766)	0.841	0.889 (0.346)	0.684
DRB3*2201	0.325 (0.569)	1.432	0.653 (0.419)	1.480	3.419 (0.064)	2.191
DRB3*0101	**12.115 (<0.001)**	**0.224**	2.919 (0.088)	0.502	0.017 (0.896)	0.980
DRB3*1101	0.460 (0.498)	0.651	**9.259 (0.002)**	**0.271**	1.368 (0.242)	0.568
DRB3*1501	0.442 (0.506)	1.785	1.435 (0.231)	2.190	0.411 (0.522)	1.489
DRB3*5702	0.061 (0.804)	0.976	0.016 (0.898)	0.950	0.230 (0.632)	1.390
DRB3*0501	0.156 (0.693)	0.724	0.120 (0.729)	1.324	0.461 (0.497)	0.681
DRB3*1601	0.007 (0.930)	0.825	0.906 (0.341)	1.945	0.369 (0.544)	0.688
DRB3*0201	1.474 (0.225)	0.460	0.862 (0.353)	2.129	0.003 (0.951)	0.907
DRB3*0701	3.125 (0.077)	-	1.792 (0.181)	3.219	0.019 (0.888)	0.813
DRB3*4401	3.125 (0.077)	-	0.618 (0.432)	1.970	0.128 (0.720)	1.403
DRB3*2601	1.081 (0.298)	3.766	0.004 (0.948)	1.231	0.033 (0.854)	0.955
DRB3*1701	1.792 (0.181)	-	0.005 (0.939)	1.190	0.581 (0.446)	0.522
DRB3*2406	**7.235 (0.007)**	**<0.00001**	0.525 (0.469)	0.220	0.631 (0.427)	-

The bold values means that is a significant association.

The BoLA-DRB3*011:01 allele (*p* = 0.002; OR = 0.271), with a frequency of 6% in the population, is associated with resistance to *B. bovis* infection. None of the alleles was associated with resistance or susceptibility to infection by *B. bigemina*.

## 4. Discussion

This is the first work on the association of BoLA-DRB3 alleles with infection by *A. marginale*, *B. bovis*, and *B. bigemina* in cattle in Brazil, as well as in the Crioulo Lageano breed.

In Creole cattle breeds, association studies between BoLA-DRB3 alleles and infectious deaseases were only carried out in the Hartón del Valle breed from Colombia using the PCR-SBT method, reporting a possible resistance alleles to leukosis (29, 30).

In the state of Santa Catarina, the highlands region has already been classified as being of enzootic instability for anaplasmosis, with a prevalence of 27.24% of positive samples in the general herd (31), including the occurrence of an outbreak of the disease in this region (32). The prevalence obtained in breeding properties of the Crioulo Lageano breed was 79.74% and the enzootic instability was not confirmed (33).

Variations in the prevalence of the disease may be associated with the time of sample collection, as the period in which the blood collections were carried out in this study, between summer and autumn, presents a higher hatching of tick larvae and, consequently, more parasitized cattle (34), favoring infection by *A. marginale, B. bovis* and *B. bigemina*. For the present study, the proportion of animals infected with *A. marginale* was 79.3%, with no animal showing clinical signs of the disease (Table 2).

The absence of regular control of vectors and the extensive rearing of animals favors constant contact with hemoparasites, reinforcing their immunity against the causative agent of the clinical disease, which would not necessarily be linked to the presence of certain alleles of the BoLA-DRB3 gene in the population since none of the sampled animals showed clinical signs.

Purebred *B. taurus* animals are more susceptible to clinical disease caused by *A. marginale* compared to animals derived from crosses between *B. taurus* and *B. indicus* and purebred *B. indicus* animals (35). This fact was not confirmed for Crioulo Lageano cattle, belonging to the *B. taurus* lineage. In addition to greater genetic diversity when compared to commercial breeds, native breeds are adapted to the specific environmental conditions of their region after than more than five centuries of natural selection and, in general, there are reports of great resistance to local pathogens (22). In Argentina, Argentine Creole cattle, also *Bos taurus*, demonstrated phenotypic resistance to *Riphicephalus (Boophilus) microplus* tick infestation in experimental infection (36). Tick resistance may also be a factor that can influence resistance to hemoparasites.

Regarding babesiosis, the data obtained for the state of Santa Catarina indicated that the prevalence of *B. bovis* in the general herd is 29.57%, while *B. bigemina* presented a prevalence of 16.73% (31). The occurrence obtained for these agents in this study was much higher for the Crioulo Lageano breed (69.2% for *B. bovis* and 61% for *B. bigemina*) but none of the animals showed clinical signs of the diseases. Despite the use of the same methodology of this study, this difference may have been due to the heterogeneity of the herd analyzed in the work by Vieira et al. (31), which, when analyzing the general herd in the state, obtained samples of *B. taurus* and *B. indicus* animals. Furthermore, this variation may also be associated with the time of sample collection, since between summer and autumn, the period in which blood samples were collected for this study, there is a greater hatching of tick larvae and, consequently, more parasitized cattle (34).

Importantly, not all the genes involved in the expression of resistance to babesiosis are known, but it is known that it is a hereditary characteristic (37) and that *B. taurus indicus* animals tend to be more resistant to babesiosis and their vectors than *B. taurus taurus* animals (38, 39). However, Crioulo Lageano animals, even those belonging to the *B. taurus taurus* lineage, do not develop the clinical disease although a large number of animals carry the agent.

In terms of possible genetic resistance to these diseases, the BoLA-DRB3*011:01 allele is frequently found in Creole breeds and has already been associated with resistance to *B. bigemina* infection (40), which was not confirmed in the Crioulo Lageano breed although it is an allele with high frequency in the population. The difference in the methodology used to identify the alleles, as well as the proportion of infected animals in this study, may justify the association or not of the allele with the disease.

Duangjinda et al. (26) identified the following resistance alleles after genotyping by the PCR-RFLP method: the *14 and *41 alleles for *A. marginale*; the *14 allele for *B. bovis*; and the *10 and *51 alleles for *B. bigemina*. We reiterate the fact that PCR-RFLP has lower precision than the PCR-SBT method and some patterns found may not correspond exactly to the expected allele. In addition, different BoLA-DRB3 alleles share the same PCR-RFLP defined variants. For this reason, results can not be compared.

An association could be established between the alleles BoLA-DRB3*001:01 and BoLA-DRB3*024:06 and *A. marginale* infection in the Crioulo Lageano breed. Similarly, the BoLA-DRB3*011:01 allele was associated with resistance to *B. bovis* infection. None of the alleles was associated with resistance to infection by the agent *B. bigemina*. The BoLA-DRB3*001:01 allele has been associated with mastitis resistance in Holstein cattle (41), while the BoLA-DRB3*011:01 allele has been associated with resistance to *B. bigemina* and also to resistance to bovine leukosis in the Hartón del Valle breed (29, 40), in addition to being associated with resistance to mastitis (41). The BoLA-DRB3*024:06 allele has previously been described in African breeds, including Sudanese breeds (42) but has not yet been associated with any type of disease.

Research has shown that Crioulo Lageano cattle are more resistant to botflies and ticks than Aberdeen Angus cattle (43). These findings suggest that factors beyond genetics play a role in determining susceptibility to diseases like anaplasmosis and babesiosis. Other factors, such as lineage, contact with vectors, and environmental adaptation, must also be considered alongside genetics.

In addition to the resistance of animals to the infection itself by the agents of anaplasmosis and babesiosis, resistance to the disease vector may also be occurring. BoLA-DRB3 alleles have already been associated with resistance to the tick *Rhipicephalus* (*Boophilus*) *microplus* (44), which would directly influence the acquisition of the infection by the animals.

Data referring to resistance alleles can help animal breeding programs. However, regarding the hemoparasites explored in this study, it is important to maintain susceptible individuals in herds to keep the stability of agents in the population and the heterozygosity of individuals, which is essential to increase the immune response to different types of infection.

## 5. Conclusion

The presence of alleles associated with resistance to *A. marginale* and *B. bovis* in Crioulo Lageano cattle highlights the importance of maintaining herd variability and heterozygosity. Further research should investigate the relationship between genetic findings and environmental and management factors that contribute to resistance to infectious disease in Crioulo Lageano animals.

## Data availability statement

The raw data supporting the conclusions of this article will be made available by the authors, without undue reservation.

## Ethics statement

The animal studies were approved by CETEA - Comitê de Ética em Experimentação Animal Universidade do Estado de Santa Catarina–UDESC. The studies were conducted in accordance with the local legislation and institutional requirements. Written informed consent was obtained from the owners for the participation of their animals in this study.

## Author contributions

MC: Data curation, Formal Analysis, Investigation, Project administration, Writing – original draft. GG: Writing – review & editing, Data curation, Formal Analysis. GF: Data curation, Writing – review & editing. EM: Data curation, Writing – review & editing. CV: Resources, Writing – review & editing. LM: Resources, Writing – review & editing. S-NT: Formal Analysis, Writing – review & editing. JF: Conceptualization, Funding acquisition, Project administration, Writing – review & editing.

## Funding

The author(s) declare financial support was received for the research, authorship, and/or publication of this article. This study was supported by the *Fundação de Amparo à Pesquisa e Inovação do Estado de Santa Catarina* (Foundation for Research and Innovation Support of Santa Catarina State; FAPESC – TERM OF GRANT SPONTANEOUS DEMAND – PESQUISA 2015; No.: 2015TR1543; PROCESSO No.: FAPESC1827/2015) and the Graduate Support Program, partially financed by the Coordination for the Improvement of Higher Education Personnel – Brazil (CAPES – Finance Code 001) for providing postgraduate fellowships and financing the translation service by the Graduate Support Program (PROAP) and Conselho Nacional de Desenvolvimento Científico e Tecnológico (CNPq), chamada CNPq Nº 4/2021 - Bolsas de Produtividade em Pesquisa - PQ - 2021 (315577/2021-5).

## Conflict of interest

The authors declare that the research was conducted in the absence of any commercial or financial relationships that could be construed as a potential conflict of interest.

## Publisher’s note

All claims expressed in this article are solely those of the authors and do not necessarily represent those of their affiliated organizations, or those of the publisher, the editors and the reviewers. Any product that may be evaluated in this article, or claim that may be made by its manufacturer, is not guaranteed or endorsed by the publisher.

## Footnotes

^1^https://www.ebi.ac.uk/ipd/mhc/
